# An Unusual Presentation of Pelvic Leiomyomatosis Misdiagnosed as Disseminated Malignancy

**DOI:** 10.1155/2012/394106

**Published:** 2012-11-11

**Authors:** Nisha Marwah, Amrita Duhan, Garima Aggarwal, Rajeev Sen

**Affiliations:** Department of Pathology, Pt. B.D. Sharma PGIMS Rohtak, BPS Government Medical College for Women (Sonepat) 1, Haryana, Rohtak 124001, India

## Abstract

Leiomyomatosis peritonealis disseminata (LPD) is an exceedingly rare, usually benign condition that clinically simulates a disseminated malignancy, occurring predominantly in women of childbearing age. We present a case of LPD in a postmenopausal woman who had undergone hysterectomy 8 years back for fibroids along with simultaneous presence of pelvic metastasis from breast carcinoma.

## 1. Introduction

Leiomyomas represent the most common gynecologic and uterine neoplasms. Approximately 20%–30% of women older than 35 years have uterine leiomyomas that present clinically. However, leiomyomas occasionally occur with unusual growth patterns or in unusual locations that make their identification more challenging both clinically and radiologically. Examples of leiomyomas with an uncommon growth pattern include diffuse peritoneal leiomyomatosis, intravenous leiomyomatosis, benign metastasizing leiomyomas, retroperitoneal leiomyomas, and parasitic leiomyomas [1]. Disseminated peritoneal leiomyomatosis is a rare condition characterized by presence of multiple smooth muscle, myofibroblastic and fibroblastic nodules on peritoneal surface of pelvic and abdominal cavity in woman of reproductive age [2].

We present a case of leiomyomatosis peritonealis in a postmenopausal woman involving the pelvic peritoneum extensively with history of hysterectomy 8 years back.

## 2. Case Report

**Figure 1:**
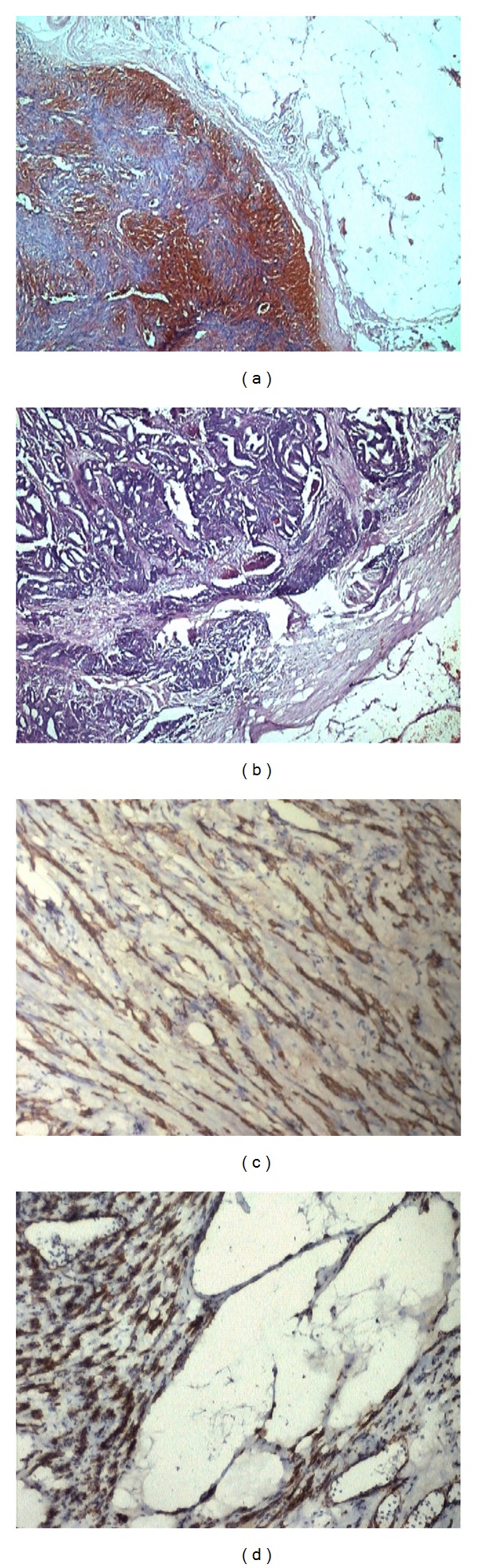
(a) Photomicrograph showing leiomyomata with retrogressive changes (H and E; ×40). (b) One mass revealing metastasis from adenocarcinoma (H and E; ×40). (c) Leiomyoma–positive for Desmin (×100). (d) Leiomyoma positive for smooth muscle actin (×100).

A 55-year-old, postmenopausal hypertensive woman presented with pelvic pain in surgery outpatient department. Her history dated back to year 2002 when she had a total hysterectomy with bilateral salpingo-oophorectomy for uterine fibroid at a private hospital. Her follow up was uneventful until next 3 years when she was operated for a pelvic tumor in a private hospital, which was locally adherent and was resected with sleeve resection of rectum, bladder, and vaginal vault. On histopathology, gross inspection revealed 3 well encapsulated masses largest measuring 17 cm, and the other two measuring 2 cm in diameter. Cut section was partly solid and partly cystic with solid areas revealing areas of whorling while the cystic component was filled with mucoid material. The histopathological impression was leiomyomata in pelvic cavity, with focal cystic and hyaline degeneration. She was again disease free for the next 3 years, then she was diagnosed with and operated for infiltrating duct carcinoma breast, positive for hormone receptors, estrogen and progesterone, while negative for Her 2/neu. In 2011 she again presented with lower abdominal pain. Her ultrasonography abdomen showed hypoechoic lesions of 3.77 × 2.43 cm in size in right iliac fossa and 2.04 × 1.28 cm in left iliac fossa. Another hypoechoic lesion 3.21 × 1.81 cm anterior to urinary bladder was also seen. On contrast enhanced computed tomography (CECT) two enlarged lymph nodes were seen in right pelvis, one anterior to bladder and one in left pelvis measuring 28 × 20 mm and 31 × 30 mm in size, respectively. Liver revealed mild fatty change. Gall bladder, pancreas, spleen, and bilateral kidneys were normal. No free fluid was present. Uterus was not visible. Her coagulation profile, liver function test, serum biochemistry, and bone scan were normal. She was nonreactive for HCV, HIV, and HBsAg. Her CA 125 level was raised to about 4 times (170 units/mL). An abdominal laparotomy was done from right and left pelvic cavities. On pathological examination, they turned out to be leiomyomata with retrogressive changes. They were positive for smooth muscle actin and desmin. Eight lymph nodes isolated showed reactive hyperplasia. However, to our surprise, one mass dissected from right pelvic cavity revealed metastasis from adenocarcinoma (Figure 1). She has been on regular follow up since then, which has been largely unremarkable.

## 3. Discussion

Leiomyomatosis peritonealis disseminata (also called diffuse peritoneal leiomyomatosis) is a rare, benign entity characterized by presence of innumerable smooth muscle nodules throughout the peritoneal cavity. It occurs mostly in women of reproductive age group who have uterine leiomyomas [3].

Although the first case was reported in 1952 by Wilson and Peale, LPD was first clearly delineated and named by Taubert et al. in 1965. In 64 reports on LPD, 105 patients have been described and amongst them only 4 were postmenopausal [4]. Most times, the patients are asymptomatic, and the disease is incidentally found, so there is a possibility that the disease incidence is higher than the number of cases described in the literature [5].

The basic pathogenesis of LPD is a multicentric metaplastic change of the submesothelial connective tissue of the abdomen due to an abnormal response to ovarian hormonal stimulation, be it normal or elevated [4]. That hypothesis also might account for the association of diffuse peritoneal leiomyomatosis with endometriosis, as the subcoelomic mesenchyme is thought to be capable of differentiating into various tissues, including endometrial glandular epithelium [1]. According to some authors, exposure to estrogen is the primary mechanism involved in the development of leiomyomatosis peritonealis disseminata. Two cases have been reported associating the disease with the utilization of tamoxifen for the management of breast cancer which might have been the causal factor in our case [5].

Finding on laparotomy overlaps with the appearances of peritoneal carcinomatosis, malignant mesothelioma, peritoneal gastrointestinal stromal tumour, and primaryperitoneal serous carcinoma. It must be distinguished from metastatic leiomyosarcoma. It has been suggested that leiomyomatosis peritonealis disseminata should be considered as diagnosis during surgery when patient has coexisting leiomyoma or diffuse leiomyomatosis of uterus with no omental caking or ascites [3].

Sometimes the imaging diagnosis may be difficult, considering that radiological findings may suggest the presence of a malignant condition [5]. The tiny peritoneal nodules of disseminated peritoneal leiomyomatosis may also be below the resolution of all radiologic techniques. When the disseminated peritoneal leiomyomatosis nodules are of sufficient size, approximately 6 mm or larger, 18F-FDG PET/CT may be used to distinguish isometabolic activity of disseminated peritoneal leiomyomatosis from hypermetabolic uptake of leiomyosarcoma. Nevertheless, direct sampling is required for definitive diagnosis and to exclude malignancy [6]. Our case is exceptional since the patient had undergone hysterectomy 8 years back. The presence of high level of CA 125 was further confusing. A diagnosis could not be made radiologically, therefore patient underwent laparotomy.

A conservative approach is recommended for this benign condition. However, chances of its malignant transformation are more compared to solitary leiomyoma, as suggested by recent reports. Hence, aggressive surgery is advocated as the first line of treatment. When surgical castration is not possible for age or desire for children, a close follow up is recommended [3].

In conclusion, the important practical issue with LPD is the potential misdiagnosis of disseminated malignancy. Given its low incidence and possible unfamiliarity to the medical community, LPD is not often considered in the differential diagnosis of multiple peritoneal nodules. On several occasions, unnecessary radical resections have been performed for this condition, with life threatening results.
